# Supplementary material for: Osteogenic Efficacy of Human Trophoblasts-Derived Conditioned Medium on Mesenchymal Stem Cells
Source: Int J Mol Sci. 2022 Sep 5;23(17):10196. doi: 10.3390/ijms231710196 (PMC9456271; doi:10.3390/ijms231710196)
Supplement: Supplementary file 1 [file ijms-23-10196-s001.zip › ijms-1896591-supplementary.pdf]

Supplementary

Table S1. Sequences of oligonucleotide primers for quantitative RT-PCR.

Primer	Direction	Sequence
IBSP	forward	GAACAAGGCATAAACGGCACC
	reverse	TTCTGCATTGGCTCCAGTGAC
ALP	forward	ACGAGCTGAACAGGAACAACGT
	reverse	CACCAGCAAGAAGAAGCCTTT
RUNX2	forward	GACACCACCAGGCCAATC
	reverse	AGAACAAGGGGGCCGTTA
Osterix	forward	GCCAGAAGCTGTGAAACCTC
	reverse	TGATGGGGTCATGGTGTCTA
OCN	forward	TGACGAGTTGGCTGACCA
	reverse	AGGGTGCCTGGAGAGGAG
OPN	forward	AGGCATCACCTGTGCCATAC
	reverse	GATGGGTCAGGGTTTAGCCA
PPAR-γ	forward	GCTGTGCAGGAGATCACAGA
	reverse	GGGCTCCATAAAGTCACCAA
Adiponectin	forward	CCTAAGGGAGACATCGGTGA
	reverse	GTAAAGCGAATGGGCATGTT
Leptin	forward	GGCTTTGGCCCTATCTTTTC
	reverse	CCAAACCGGTGACTTTCTGT
BMP2	forward	GGACATGGTTGTGGAGGGTT
	reverse	AAAGTGTCAACTGGGGTGGG
GREM	forward	AACAGTCGCACCATCATCAA

	reverse	CGATGGATATGCAACGACAC
CXCL1	forward	AAGTGTGAACGTGAAGTCCC
	reverse	GTCACTGTTCAGCATCTTTTCG
CXCL3	forward	TGGTCACTGAACTGCGCT
	reverse	ATGCGGGGTTGAGACAAG
CXCL6	forward	AGAGCTGCGTTGCACTTGTT
	reverse	GCAGTTTACCAATCGTTTTGGGG
CXCL8	forward	ACTGAGAGTGATTGAGAGTGGAC
	reverse	ACAACCCTCTGCACCCAGTT
CCL7	forward	AGAGCTGCGTTGCGTTTGT
	reverse	CTATGGCGAACACTTGCAGATTAC
CCL11	forward	TCTGTGCCTGCTGCTCATAG
	reverse	TGGAATCCTGAACCCACTTC
CCL13	forward	GTCCCCAGAAGGCTGTCATC
	reverse	GGACCCACTTCTCCTTTGGG
IL-6	forward	TCTTCAGAACGAATTGACAAACAAA
	reverse	GCTGCTTTCACACATGTTACTCTTG
MMP-1	forward	TTGAGAAAGCCTTCCAACTCTG
	reverse	CTGCAACACGATGTAAGTTGTA
MMP-3	forward	TGCTGCTCATGAAATTGGCC
	reverse	TCATCTTGAGACAGGCGGAA
MMP-8	forward	TCTGCAAGGTTATCCCAAGG
	reverse	CTTGCTGGAAAACTGCATCA
MMP-13	forward	AGCACCCTTCTCATGACCTC

	reverse	TCTTTTGGAAGACCCAGTTCA
GAPDH	forward	TCGCCCCACTTGATTTTGG
	reverse	GCAAATTCCATGGCACCGT

Table S2. Antibodies used in western blot analysis.

Antibodies	Host	Manufacturer	Catalog #	Category	Dilution
ERK1/2	Rabbit	Cell Signaling	9102S	Primary	1:1000
p-ERK1/2	Rabbit	Cell Signaling	9101S	Primary	1:1000
p38	Rabbit	Cell Signaling	9212S	Primary	1:1000
p-p38	Rabbit	Cell Signaling	9211S	Primary	1:1000
JNK	Rabbit	Cell Signaling	9252S	Primary	1:1000
p-JNK	Rabbit	Cell Signaling	9251S	Primary	1:1000
AKT	Mouse	Cell Signaling	2920S	Primary	1:1000
p-AKT	Rabbit	Cell Signaling	9271S	Primary	1:1000
β-actin	Mouse	Santa Cruz	sc4778	Primary	1:200
Mouse IgG-HRP	Goat	Abcam	ab6789	Secondary	1:5000
Rabbit IgG-HRP	Goat	Abcam	ab6721	Secondary	1:5000

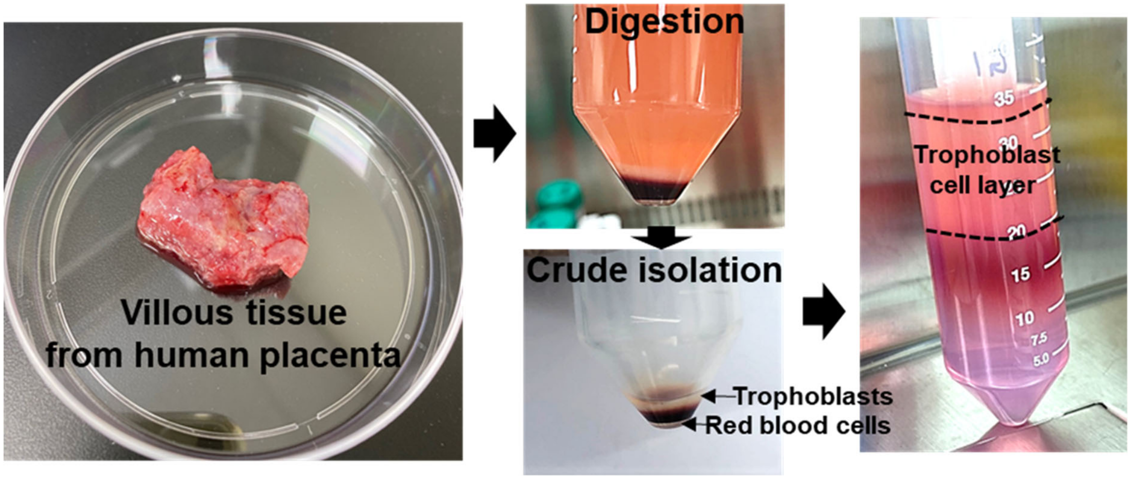

Supplementary Figure S1. Isolation procedure of primary trophoblasts from the human placenta.

Villous tissue isolated from the human placenta was digested with DNase and trypsin. Supernatants from the digestion process were collected and then centrifuged with fetal bovine serum. Crude trophoblasts were observed between the FBS and RBC (red blood cells) layers. They were subsequently obtained by density gradient centrifugation.

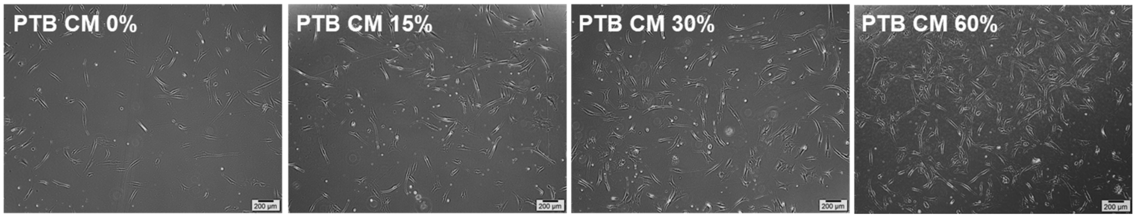

Supplementary Figure S2. Proliferation effect of trophoblast-derived conditioned medium (TB-CM).

Phase-contrast images of mesenchymal stem cells treated with 0, 15, 30, and 60% TB-CM for 24 h.
